# Amniotic Fluid Stem Cells: A Novel Source for Modeling of Human Genetic Diseases

**DOI:** 10.3390/ijms17040607

**Published:** 2016-04-22

**Authors:** Ivana Antonucci, Martina Provenzano, Melissa Rodrigues, Andrea Pantalone, Vincenzo Salini, Patrizia Ballerini, Cesar V. Borlongan, Liborio Stuppia

**Affiliations:** 1 Department of Psychological, Health and Territorial Sciences, School of Medicine and Health Sciences, G. d’Annunzio University, Chieti-Pescara, Via dei Vestini 31, 66013 Chieti, Italy; i.antonucci@unich.it (I.A.); martina.provenzano@unich.it (M.P.); melissa.rodrigues@unich.it (M.R.); p.ballerini@unich.it (P.B.); 2 Ce.S.I-Met, G. d’Annunzio University, Chieti-Pescara, Via Colle dell’Ara n.1, 66100 Chieti, Italy; 3 Department of Medicine and Aging, G. d’Annunzio University, Chieti-Pescara Via dei Vestini 31, 66013 Chieti, Italy; pantaloneandrea@libero.it (A.P.); vincenzo.salini@unich.it (V.S.); 4 Department of Neurosurgery and Brain Repair, Center of Excellence for Aging and Brain Repair, University of South Florida College of Medicine, Tampa, FL 33612, USA; cborlong@health.usf.edu

**Keywords:** amniotic fluid stem cells, pluripotency, modeling of genetic diseases, drug testing, trans-generational epigenetic modifications

## Abstract

In recent years, great interest has been devoted to the use of Induced Pluripotent Stem cells (iPS) for modeling of human genetic diseases, due to the possibility of reprogramming somatic cells of affected patients into pluripotent cells, enabling differentiation into several cell types, and allowing investigations into the molecular mechanisms of the disease. However, the protocol of iPS generation still suffers from technical limitations, showing low efficiency, being expensive and time consuming. Amniotic Fluid Stem cells (AFS) represent a potential alternative novel source of stem cells for modeling of human genetic diseases. In fact, by means of prenatal diagnosis, a number of fetuses affected by chromosomal or Mendelian diseases can be identified, and the amniotic fluid collected for genetic testing can be used, after diagnosis, for the isolation, culture and differentiation of AFS cells. This can provide a useful stem cell model for the investigation of the molecular basis of the diagnosed disease without the necessity of producing iPS, since AFS cells show some features of pluripotency and are able to differentiate in cells derived from all three germ layers “*in vitro*”. In this article, we describe the potential benefits provided by using AFS cells in the modeling of human genetic diseases.

## 1. Background

Despite continuous increase in our knowledge about the genetic basis of a number of congenital and late-onset human diseases, so far a large majority of these conditions still remain untreatable. This is largely due to the lack of information about the precise sequence of early molecular events occurring during tissue development and underlying the pathogenesis of the disease. Indeed, although gene mutations or chromosomal aberrations responsible for the disease are present in all cells of affected individuals, the functional damage generally involves only a few tissues (or even a single one) in which the expression of the affected gene(s) occurs, affecting normal cell function. The collection of human tissues other than blood from living patients is hampered by several factors, and in most cases, at the time of diagnosis, the disruption of the cellular and/or tissue function has already occurred, making it impossible to get a clear picture of the progressive involvement of different pathways during cell differentiation. The use of animal models for the study of the consequences of gene mutations during development, although able to provide useful information, does not produce results which can be entirely translated to humans, due to the anatomical and physiological differences between the two species [1]. Indeed, animal models often are not able to completely represent the pathological mechanisms underlying human diseases, as in the human system where the pathology and the disease are related to specific molecular pathways connecting genotype to phenotype. Human stem cells could provide very important models to understand the molecular basis of genetic diseases, to perform functional studies on their development and to identify new therapeutic approaches. The best cells for disease modeling are so far considered to be the human pluripotent stem cells, namely Embryonic Stem cells (ES) and induced Pluripotent Stem cells (iPS), harboring naturally occurring disease-causing mutations and genomic aberration [2]. Human ES cells isolated from embryos, carriers of genetic mutations as evidenced by Preimplantation Genetic Diagnosis (PGD) carried out in Assisted Reproduction Techniques clinics, could represent a source of pluripotent cells that theoretically can generate any cell type within the human body [3]. However, ES cells are the subject of ethical controversy since their first isolation in 1998 [4], and their use for scientific purposes is limited in several countries. In this view, in recent years, great interest has been devoted to the use of iPS cells for the modeling of human genetic diseases. IPS cells are the result of the original findings of Yamanaka and colleagues, who demonstrated that human fibroblasts could be reprogrammed to pluripotency by the transduction of just four transcription factors [5]. This discovery represented a breakthrough in the study of genetic diseases, making it possible to collect fibroblast carriers of the causative genetic alteration from the affected patients, to reprogram these cells to pluripotency and to induce specific differentiation into the affected lineages in order to reveal aberrant phenotypes in culture [3]. Numerous genetic diseases have been so far analyzed by using the iPS cell modeling approach, thus confirming the usefulness of this tool for the study of genetic diseases [3]. Among these disorders, Down syndrome [3], Duchenne and Becker Muscular Dystrophy [3,6], Huntington Disease [3,7], Gaucher Diseases [3,8] and Fragile X syndrome [9] represent just a few examples. In some cases, the modeling of the genetic disease by iPS cells has provided novel information about the molecular mechanisms underlying the pathogenesis of the disease; noteworthy findings include the silencing of TERC locus in the dyskeratosis congenital disorder [10], the altered cellular localization of KCNQ1 in LQT syndrome [11], the reduced synaptic connectivity in Rett Syndrome [12], and the increased oxidative-stress response in Parkinson Disease [13]. However, iPS cells are not devoid of limitations, including the artificial methods involved in generating them, which leads to questions about: (i) how closely they can resemble the identity and function of both normal and disease specific differentiated adult cells; (ii) potential false positive or negative results difficult to quantify; and (iii) epigenetic memory of the deriving adult cells not always perfectly erased [14]. Moreover, based on the limited invasiveness of the procedure, iPS cells are commonly obtained from dermal fibroblasts, but the accumulation of mutations resulting from aging and UV exposure [14] as well as from karyotype abnormalities and gene mutations occurring during propagation in culture [15] may represent a further criticality. Finally, iPS cells only allow the study of non-prenatal lethal genetic mutations or genomic aberrations. In this regard, the use of alternative models for the study of human genetic diseases, able to by-pass the aforementioned criticisms, could be of huge value for increasing the usefulness of these kinds of approaches. In this review, we will analyze the model provided by Amniotic Fluid Stem cells (AFS) [16] as a novel and alternative source to be used for the investigation of the molecular basis of human genetic diseases. AFS cells have been demonstrated to offer an interesting model of stem cells, sharing some features with ES cells, and providing a useful alternative tool for examining stem cell therapy.

## 2. AFS Cells: Features and Properties

Human amniotic fluid obtained during the process of amniocentesis contains a heterogeneous cell population, originating from embryonic and extra-embryonic tissues. The properties of AFS cells vary with gestational age and different approaches have been identified to isolate and characterize these types of stem cells [17,18,19]. Based on morphological and growth characteristics, the adherent human AF cells can be classified into three main groups: epitheloid (E-type) cells, amniotic fluid specific (AF-type) cells and fibroblastic (F-type) cells. AF-type and F-type both appear at the beginning of cultivation, while E-type cells usually appear later and not in all fluid samples [20,21,22]. Approximately 1% of the cells in cultures express the membrane receptor c-kit (CD117) [16]. The first demonstration of the presence in human AF of cells expressing OCT 4 an important marker of pluripotency, was reported by Prusa *et al.* (2003) [23]. In the same year, In’t Anker *et al.* described that human AF contains a fibroblast-shaped cell population positive for mesenchymal markers, such as CD90, CD105, CD73 and CD166, but negative for the hematopoietic markers, such as CD45, CD34 and CD14 [24]. Thereafter, a complete characterization of AFS cells has been reported by De Coppi *et al.* (2007), who isolated c-Kit (CD117) positive populations with high clonogenic potential [16]. Clonal AFS cell lines show self-renewal capacity, can be expanded extensively in feeder layer-free cultures with an approximate doubling time of 36 hours, and, more interestingly, maintain a constant telomere length for over 250 doublings [16]. Importantly, despite their high proliferation rate, AFS cells preserve a constant morphology, apoptosis rate and marker expression of pluripotency up to 25 passages [25]. *In vitro* experiments have demonstrated the ability of these cells to differentiate into all three germ layers giving rise to adipogenic, osteogenic, myogenic, endothelial, neural and hepatic cells, under appropriate culture conditions [16,26,27,28,29]. In view of these considerations, AFS cells have been classified as a novel type of broadly multipotent stem cells sharing characteristics of both embryonic and adult stem cells [16,30]. Unlike ES, AFS cells do not form teratomas after transplantation in nude mice [16] and are considered as ideal candidates for therapeutic applications, circumventing any ethical objections, given that amniocentesis is a widely accepted procedure for prenatal diagnosis. Interestingly, it has been reported that human AFS cells could be efficiently infected by first generation adenovirus vectors, and infection and expression marker genes have no effect on the cells phenotype and differentiation potential, suggesting that adenovirus may be useful to engineer AFS cells which may be used in a wide range of gene therapy treatments [31].

To date, several protocols have been used for the isolation and differentiation of AFS cells. Although the majority of studies are based on c-Kit selected cells [16,32], other groups have directly cultured unselected AFS cells in media allowing their proliferation and differentiation [26,33,34,35]. An important point here is to determine if specific properties concerning the stemness and differentiation ability of unselected AFS cells are identical or different to those of c-Kit^+^ AFS cells*.* Based on reports, there is scientific evidence that c-Kit^+^ and unselected AFS cells show similar but not identical properties and are both able to produce lineages representative of the three germ layers [21,36,37]. Furthermore, cultured human AFS cells, in particular the unselected ones, express a wide range of pluripotency markers, such as OCT4, SOX2, SSEA4, SSEA3, c-MYC, KFL4 [38] and differentiation markers including BMP-4, nestin, AFP, HNF-4α and GATA 4. Most importantly, the immunomodulatory capacity and low immunogenicity of these cells makes them promising candidates for allogeneic transplantation and clinical applications in regenerative medicine. Along this view, several studies have reported that AFS cells are positive for antigens HLA-ABC (MHC class I), but only a small fraction are slightly positive for antigens HLA-DR (MHC class II) [16,39]. In addition, these cells appear resistant to rejection because they express immunosuppressive factors such as CD59 (protectin) and HLA-G [39]. Recently, a number of studies have suggested the paracrine potential of these cells and their secretome is being considered as an important source of cytokines, chemokines and pro-angiogenic soluble factors, such as monocyte chemoattractant protein-1 (MCP-1), stromal cell-derived factor-1 (SDF-1) and VEGF [40,41,42]. The paracrine effect was demonstrated *in vivo* in a rodent model of ischemic stroke, where transplantation of human AFS cells facilitated a reduction of the injured area, together with increment of endogenous cell proliferation and subsequent differentiation into neuronal lineage in the host brain [43,44]. Of particular interest, the conditioned medium of AFS cells is able to exert a remarkable pro-survival and anti-apoptotic effect on preclinical models of acute myocardial infarction [45]. The secretion of cardioprotective and proangiogenic factors decreased the infarct size and cardiomyocyte death within two hours by treatment. In light of these results, the isolation and administration of specific stem cell-derived paracrine factors may represent a promising therapeutic approach for the treatment of cardiovascular disease, and, in particular, new cardioprotective molecules could be identified and used in future clinical studies. In this scenario, AFS cells may be considered as an ideal candidate for paracrine therapy, and their secretome could be used for regenerative medicine applications.

In the last few years, several groups reported that both first and second trimester AFS cells (human CD117^+/−^ selected) show common characteristics with primordial germ cells (PGC) including expression of Fragilis, Stella, Vasa, c-Kit, Rnf17, DAZAL, thus suggesting a possible origin of AFS cells from epiblast-derived cells such as PGC or PGC progenitors [38]. Based on these evidences, it can be hypothesized that a small percentage of PGCs are likely lost in the AF, and this could explain the presence of early markers of germ cells in the AFS cell population [46,47]. Nevertheless, further studies are required to support this hypothesis and the debate on the origin of these cells is still open.

## 3. Are AFS Cells Pluripotent Stem Cells?

AFS cells may correspond to a new class of stem cells with properties of plasticity intermediate between embryonic and adult stem cell types in terms of their versatility [16,48]. In the past, some researchers have classified AFS cells as human pluripotent stem cells (hPS) [23,49,50] but this claim is debatable for two important reasons: (1) AFS cells do not form tumors *in vivo*; (2) so far there is no data on their ability to produce chimeras when injected into blastocysts. Since the unique abilities of hPS consist of self-renewal, differentiation into derivatives of all three germ layers, and formation of clonal lines *in vitro* and teratomas *in vivo*, we can conclude that AFS cells are not true hPS. However, AFS cells display molecular and biological characteristics closer to hPS than to multipotent stem cells, expressing markers of pluripotency (OCT4, NANOG, SOX2 and c-MYC, KFL4) both at the mRNA and protein level and being able to form monoclonal lines capable of differentiating in distinct lineages representative of the three germ layers. In 2010, for the first time, it was reported that clonal human c-Kit^+^ AFS cells have the ability to form embryoid bodies (EB) just as ES cells and PGCs [51]. EB are three-dimensional cell aggregates that recapitulate the initial steps of early mammalian embryogenesis and represent a crucial stage in the differentiation of hPS into the three germ layers. Similar to ES cells, EB generation by AFS cells is regulated by the mTor pathway [51] and only in 2012, Moschidou *et al.* demonstrated that first trimester human c-Kit^+^ AFS cells are able to form beating EB with high efficiency [52]. In this regard, our group demonstrated the ability of unselected second trimester AFS cells to form *in vitro* EB with pluripotency potential and features of early stage embryogenesis [38]. These considerations suggest that AFS cells could provide an alternative source to hPS for their valuable features: (a) high accessibility by means of routine amniocentesis; (b) the ability to differentiate in lineages representative of the three germ layers; (c) the capacity to form EB and; (d) the therapeutic safety.

A comparison of the main properties and differences between ES, iPS and AFS cells is summarized in Table 1.

## 4. Reprogramming AFS Cells from Human Disease

Due to the previously described limitations of iPS cells in the modeling of human genetic diseases, AFS cells have been thought to be a viable alternative to the use of human ES and iPS. Indeed, these cells could represent a good resource for disease modeling and, consequently, one of the most promising tools in medical genetics. AFS cells can be isolated from the AF of women undergoing prenatal diagnosis to assess the presence of genomic aberrations or genetic mutations in the fetus. In the last few years, the proportion of advanced maternal age pregnancies has been rising worldwide due to changes in lifestyle; as a consequence, the number of fetuses carrying chromosomal aberrations, such as trisomy 21, is unfortunately increasing. On the other hand, the development of non-invasive prenatal diagnosis techniques, such as the analysis of cell-free DNA in maternal blood, together with the improvement of the standard first-trimester screenings, such as nuchal translucency and biochemical analysis, are allowing for a better selection for women undergoing amniocentesis allowing the isolation of an increased number of AFS cell lines carrying abnormalities. Furthermore, since AFS cells can be easily reprogrammed through a wide variety of methods [52,53,54,55,56,57,58,59], they are able to erase the epigenetic memory after reprogramming [54] and can be easily differentiated into different cell types. Recently, Moschidou *et al.* showed that human 1st trimester AFS cells can be fully reprogrammed to pluripotency with non-viral methods and non-integrating systems, solving the problems related to genome integration of transgenes and the potential risk of virally induced tumorigenicity [52]. In this view, AFS cells could provide an alternative source of pluripotent cells and in the future could be utilized in a clinical setting.

**Figure 1:**
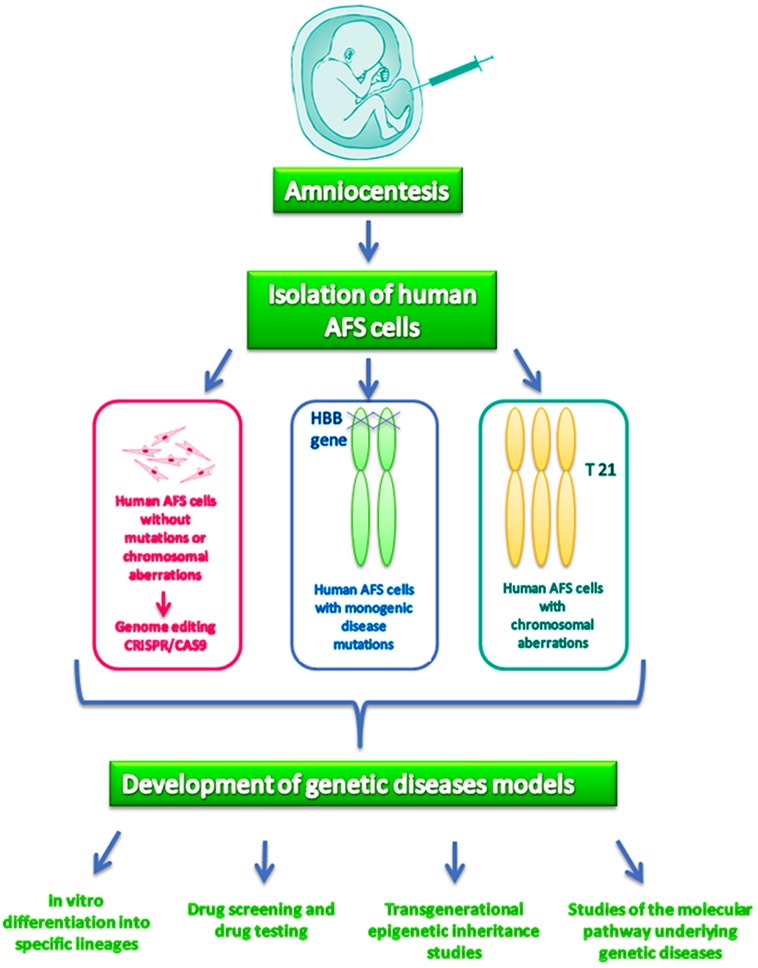
AFS cells in the study of human genetic diseases.

A few examples of this possibility have already been reported in the literature. Fan and colleagues, in 2012 [57], generated β-thalassemia homozygous iPS cells both from AFS cells and from skin fibroblasts using a doxycycline-inducible lentiviral system to compare the efficiency of both cellular models. Interestingly, the obtained results demonstrate that human β-thalassemia iPS can be more rapidly and efficiently generated from AFS cells than from adult skin cells, opening up the possibility of testing mutation-specific drugs that could be used for the early perinatal treatment of the affected newborn. AF-iPS have also been generated from human second trimester fetuses with trisomy 21 obtaining an *in vitro* model of Down syndrome allowing the modeling of the neurogenesis in AF-iPS with trisomy 21 and highlighting the role of miR-155 and miR-802, both encoded by chromosome 21, in the impairment of neuronal differentiation [28,60]. Moreover, trisomy 21 AF-iPS cells showed a significantly lower efficiency in neuronal differentiation than normal AF-iPS, suggesting that the overexpression of APP is a factor leading to impairments during neurogenesis. In light of these premises, AFS cells represent a promising tool to model *in vitro* pathogenic phenotypes, and the establishment and banking of clonal AFS cell lines derived from pregnancies with specific genetic aberrations would be of highest value for basic research and drug discovery [61] (Figure 1). However, a limitation in this approach is that gene modeling by AFS cells would involve only genetic diseases currently investigated in prenatal diagnosis, such as chromosomal abnormalities or monogenic disease in at risk families, but not multifactorial disease or late onset monogenic diseases. However, in recent times, the emerging technology for genome editing, also known as CRISPR (Clustered Regularly Interspaced Short Palindromic Repeats)/Cas9 system, has allowed the generation of disease models for both monogenic and complex genetic disorders, enabling creation of knockout cells *in vitro*. Preliminary results of our group suggest that AFS cells are capable of generating human cellular disease models *in vitro* by CRISPR*/*Cas9 system with great speed and efficiency (data not shown).

## 5. AFS Cells and Drug Testing

Drug development is a costly and long multi-phase process burdened with a high rate of failure mainly occurring in late phase III trials or at registration. This happens in a scenario in which both libraries containing thousands of testable compounds with the potentiality to become therapeutic agents and technological platforms for high-throughput screening are dramatically improved. One recognized reason for the high rate of failure of many drug candidates is the poor predictability of the preclinical studies carried out on animal models, which are mined by significant species-specific differences in the genetic background and in the mechanisms underlying the disease etio-pathology. The use of *in vitro* cell cultures provides an efficient assay to individuate a selected list of promising drug candidates for further studies. The results, aimed to verify the efficacy and safety of the compounds towards a disease rely on the possibility to perform the appropriate tests on human disease-specific primary cell targets identified as “physiologically-relevant cells”. With respect to the engineered cell lines, human primary cells have the advantage that the drug targets are evaluated in their unaltered biological environment being regulated by wild type, native elements. However, physiologically-relevant primary cells show also several significant limitations, including low proliferation rate, unstable *in vitro* phenotype, batch-to-batch functional variability and difficulty in tissue source accessibility (e.g., neuronal cells, pancreatic β cells, hepatocytes) [62]. The advent of iPS technology has advanced an alternative model to physiologically-relevant primary cells [5], allowing access to different disease-relevant targets useful for high throughput pharmaceutical drug screening in the spirit of the 3Rs principles aimed at reducing, refining and replacing the use of animals in all areas of drug developmental processes [63]. The modeling of human genetic diseases by iPS cells allowed to test some specific drugs and to evaluate their efficiency on the derived cellular model, such as in the case of valproic acid and tobramycin in Spinal Muscular Atrophy [64], isoproterenol, cisapride and nifedipine in LQT syndrome [65], IGF1 and gentamicin in Rett Syndrome [12]. Moreover, human iPS cells have also been used, although to a lesser extent than originally anticipated, in toxicity drug screening, mainly aimed at evaluating cardiotoxicity and hepatotoxicity, which represent some of the major toxicological concerns in drug developmental processes. Indeed, human iPS cells have been differentiated into cardiomyocytes and hepatocytes, with genotypic and phenotypic characteristics that enable them to exhibit relevant drug responses [66]. Increasing evidences also indicate that human iPS cells can be successfully differentiated into neurons with *in vivo*-like properties that make them suitable to be used in both targeted and phenotypic/physiological pharmacological screening as well as in drug neurotoxicity evaluation [67]. Could AFS cells represent an alternative tool to iPS for drug discovery and safety assays as well? The absence of sufficient experimental data in literature does not allow drawing conclusions on this point. However, a very interesting model is suggested by recent reports showing that human AFS cells could represent precursors of gametes [38,68], which usually cannot be investigated in humans due to their development only in the post implantation stage of embryo development. Thus, the use of AFS cells as a model of germ cell precursors would allow investigating the mechanisms underlying drug-induced effects in gametogenesis and to screen either natural or synthetic compounds potentially useful to preserve fertility, without interfering with the efficacy of chemotherapy or other treatments. This model could also be very useful in studying the effects of additional substances, for example, Δ^9^tetrahydrocannabinol which accounts for the majority of the reproductive hazards in marijuana preparations [69].

In relation to their utility for evaluating drug cardiotoxicity and hepatotoxicity, studies have also demonstrated the differentiation potential of AFS cells into cardiomyocytes and hepatocytes [19,70,71,72,73].

On the other hand, although AFS cells have been reported to promote nerve regeneration and myelination and to produce several cytokines and neuro-glial factors [74], there is still very little data demonstrating their differentiation into neurons [75], thus limiting at present their use as a model for drug neurotoxicity evaluation.

AFS cells have been also reported to form three-dimensional (3D) chimeric organoids with mouse embryonic kidney cells [76]. The human cells have been able to form glomerular structures, to differentiate into podocytes and to internalize exogenously administered bovine serum albumin, recapitulating not only their morphology, but also their peculiar functional features [76]. In contrast to traditional cell culture set-up in a dish, 3D systems enable the three-dimensional growth of cells, which in turn can develop into an organotypic structure able to exert organ-specific functions [77]. Moreover, they can also be expanded for a very long time in culture maintaining intact stem cell compartments [78]. These peculiar characteristics make this cell culture technique a promising tool not only for tissue replacement therapy, but also for drug-testing studies. In this view, a functional readout developed in human organoids aimed at facilitating drug development and personalized medicine in cystic fibrosis has been recently reported [79].

**Figure 2:**
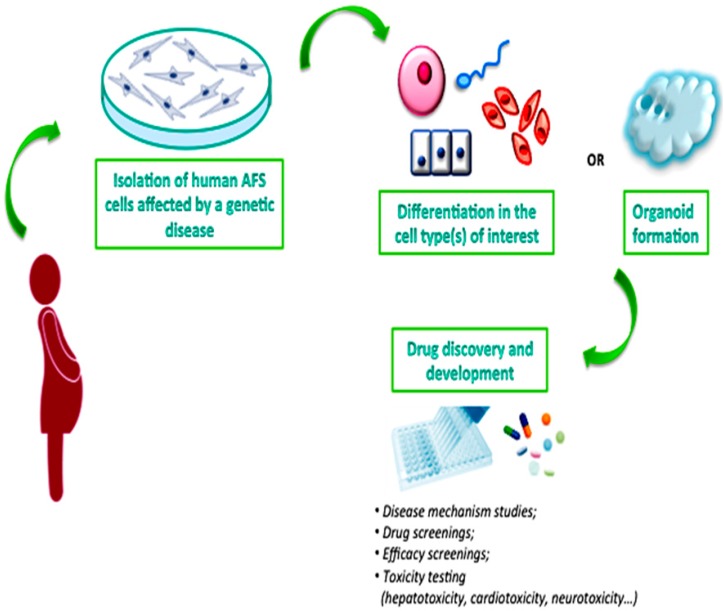
AFS cells as a novel system for drug development.

In conclusion, AFS cells, with their potential large-scale amplification, differentiation and 3D organoid formation, could represent a very interesting source to produce physiologically-relevant novel systems to adequately test pharmaceutical agents before their administration to patients, although additional experimental data that confirms this hypothesis is still required (Figure 2).

## 6. Future Perspectives: AFS Cells for the Study of Trans-Generational Effects of Epigenetic Alterations

While an organism’s genotype is static throughout its life, the epi-genome is highly dynamic and can adapt or be altered in response to the internal or external environmental factors. Germ cell development is a critical period during which epigenetic patterns are established and maintained. If epigenetic errors are introduced at any time during germ line development, some serious consequences for fertility and the health of future offspring(s) may arise [80]. The effects of developmental programming can be induced by the mother when exposed to intrauterine environmental factors during pregnancy affecting the fetus (F1 generation) but also the germ line of the fetus (F2 generation). When developmental programming is transmitted beyond F3 generations, it is considered to be trans-generational and cannot be explained by direct environmental exposure anymore. To date, only a few research groups have provided direct evidence for trans-generational epigenetic inheritance, mostly indicating transmission through the paternal line [81]. However, the majority of the reports have sparked scientific debate, and, thus, the exact reasons for this inheritance remain a mystery. Biologists first observed this “trans-generational epigenetic inheritance” in plants [82], but, over the past few years, evidence has been accumulating that the phenomenon occurs in rodents and humans as well. The first evidence in humans was found in the mid-2000s, after large epidemiological investigations in Europe began to show trans-generational effects [83]. To study the phenomenon of trans-generational epigenetics, biologists are now directing their attention to fathers, looking at how sperm might gain and lose epigenetic markers. In the past few years, several *in vivo* studies are supporting the observations previously reported in the epidemiological studies and have begun to attribute the transmission of various traits to changes in sperm [84]. Most recently, reports that the sperm of male offspring showed changes in DNA methylation persisting for at least four generations [85] sparked interest in the field. Remarkably, the exposure to chemicals like bisphenol A and vinclozolin has also shown altered behavior in F1 generation and an increased incidence of diseases three generations after the exposure, inherited via the male germ line [86]. Furthermore, some reports on prolonged drug use showed the potential to promote heritable epigenetic modifications including acetylation that could place progeny at increased risk for drug abuse later in life [87]. Despite emerging evidence of the possibility of trans-generational inheritance, these studies are limited by the challenges in obtaining and analyzing the small number of primordial germ cells, since human germ cell development is not traceable through direct analysis *in vivo* [80]. Therefore, an *in vitro* model mimicking human gamete formation would be an extremely valuable research tool. As previously described, AFS cells have been recently demonstrated to share several features with PGC, thus suggesting their use as an efficient handy tool to fulfill the scarcity of *in vitro* models to study human gametogenesis and fill the void of insufficient data on germ-line diseases and diseases caused by epigenetic alterations endangering trans-generational inheritance.

## 7. Conclusions

Based on literature data, AFS cells have been so far proposed to be an interesting model for cell therapy, due to their ability to differentiate into different cell types and their lack of ethical concerns. It is important to underline that disease modeling represents an appropriate alternative to animal experimentation because it allows overcoming all limitations related to preclinical research such as the ethical implications and excessive costs of animal models. In light of data described in the present review, it can also be hypothesized that AFS cells will become one of the main models for the modeling of human genetic diseases in the near future. The advantages of this model as compared to the iPS cell paradigm can be summarized as follows:

(1) AFS cells can be easily collected from women undergoing amniocentesis, a prenatal diagnostic tool currently used in Western countries in a growing number of pregnancies, mostly due to the increased cases characterized by advanced maternal age caused by social and cultural factors. The recent improvements in the first trimester non-invasive tests for prenatal diagnosis will likely reduce the total number of amniocentesis, but will increase the rate of genetically abnormal pregnancies, thus providing a large number of samples to be used for the modeling of specific genetic diseases;
(2) Unlike iPS, AFS cells will provide the opportunity to also investigate diseases that are lethal during pregnancy. Moreover, AFS cells are less prone to aging dependent genetic and epigenetic modifications as compared to fibroblasts, commonly used for the generation of iPS, thus providing a more natural model for the study of the disease;
(3) AFS cells can be easily reprogrammed by using both viral and non-viral methods, making this process very efficient. Moreover, the use of non-viral methods will likely reduce the cost of the reprogramming procedure as compared to the use of iPS, which also requires the availability of specific facilities for the use of viral vectors.

It can also be hypothesized that, in the coming years, the newly discovered genome editing technique, CRISPR, could provide a useful tool to increase the number of *in vitro* models, allowing the procurement of AFS clones with induced mutations and, consequently, the study of a wide range of pathogenic conditions. The recent advances of functional genetics including genome-scale loss-of-function drug-suppressor screens, obtained by the application of the CRISPR-Cas9 technique, can be successfully applied to these kinds of cells, further creating novel opportunities for testing the modes of action and the efficacy of various drug candidates.

## Figures and Tables

**Table 1:** Properties and differences between ES, iPS and AFS cells.

Properties	ES	iPS	AFS
Naturally existing stem cells	Yes	No	Yes
Self renewal capacity	Yes	Yes	Yes
High proliferation efficiency	Yes	Yes	Yes
Pluripotent marker expression	Yes	Yes	Yes
Differentiation in three germ layers	Yes	Yes	Yes
Risk for teratoma formation	Yes	Yes	No
Ectopic oncogene expression	No	Yes	No
Immunological compatibility with recipient	Yes/No	Yes/No	Yes/No
Possessing prenatally lethal mutations	Yes	No	Yes/No
Disease-specific stem cells	Yes	Yes	Yes
Disease specific stem cells with known patient’s phenotype	No	Yes	Yes
Risk for chromosomal aberrations from the donor cells	No	Yes/No	No
Risk for aberrations acquired during reprogramming	No	Yes	No
Epigenetic deregulation	No	Yes	No
Studies on drug testing	Yes	Yes	Yes
Legal restrictions	Yes	No	No
Ethical concerns	Yes	No	No
